# COVID-19 vaccination does not affect male sexual functions

**DOI:** 10.1186/s12958-022-01052-8

**Published:** 2023-01-13

**Authors:** Poonam Mehta, Arijit Chakraborty, Syed Waseem Andrabi, Bhawani Sharma, Rakesh Kumar, L. V. K. S. Bhaskar, Singh Rajender

**Affiliations:** 1 Division of Endocrinology, CSIR-Central Drug Research Institute, Lucknow, India; 2 Academy of Scientific and Innovative Research (AcSIR), Ghaziabad, India; 3 Department of Sports Physiology, National Sports University, Imphal, Manipur India; 4 Milann Fertility Centre, Greater Kailash, New Delhi, India; 5 Shri Mata Vaishno Devi University, Katra, Jammu & Kashmir India; 6 Department of Zoology, Guru Ghasidas Vishvavidyalya, Bilaspur, Chhattisgarh India

**Keywords:** COVID-19 vaccine, Male sexual functions, Erectile function

## Abstract

**Background:**

COVID-19 infection has been linked with erectile dysfunction, which has also raised apprehensions about the impact of COVID-19 vaccination on male sexual functions. The purpose of this study was to investigate the impact of COVID-19 vaccination on male sexual functions, such as erectile function, orgasmic function, sexual desire, intercourse satisfaction, and overall satisfaction.

**Methods:**

We used International Index of Erectile Function (IIEF) questionnaire for data collection. Mixed methods were adopted for this study, which consisted of Google online form distribution and the distribution of hard copies of the form to those who were not internet friendly. All data were entered in a spreadsheet and scores were assigned to each response according to the standard scores given in the IIEF questionnaire. Fifteen questions, one corresponding to each question in the IIEF questionnaire, were included to assess the impact of COVID-19 vaccination on each sexual function.

**Results:**

In the first part of analysis, we calculated sexual function scores and men reporting low sexual function scores (~ 15%) were excluded, providing us with 465 individuals for further analysis. Regarding the impact of COVID-19 vaccination on male sexual functions, 71% individuals reported no impact, 3% reported a decline, 2.7% reported an improvement, and 23.3% could not assess the impact. We also performed analysis on the basis of age-groups of the participants and the duration after vaccination, finding that there was no impact irrespective of the age of subjects or the length of period after vaccination.

**Conclusions:**

COVID-19 vaccination does not affect male sexual functions, including erectile function, orgasmic function, sexual desire, intercourse satisfaction, and overall sexual satisfaction.

**Supplementary Information:**

The online version contains supplementary material available at 10.1186/s12958-022-01052-8.

## Background

The novel Severe Acute Respiratory Syndrome coronavirus 2 (SARS-CoV-2) caused havoc during 2020-2021. It resulted in more than 6,174,969 deaths [1] and significant morbidities in even higher number of individuals worldwide. Some of the after-effects of the viral infection are still being discovered. COVID-19 affected men more often than women and various studies have presented this sex skewness in SARS-CoV-2 infection rates as well as the associated morbidities and mortality [2]. Among various factors accounting for this difference are androgens, which affect susceptibility and sensitivity to COVID-19. In this connection, the androgen receptor has been implicated in mediating the virus’s entry into cells and the process of infection [3, 4]. Eventually, the morbidity of COVID-19 differed significantly between the two sexes. Some of the morbidities might have initially gone unnoticed or remained unconfirmed due to the lack of epidemiological data. Erectile dysfunction (ED) is one among them, which was first linked with COVID-19 disease by investigations carried out at the University of Florida, USA, finding that the patients with COVID-19 were 3.3 times more likely to have erectile dysfunction, and the association remained strong even after adjusting for various confounding factors [5]. Another study in the United States on 230,517 men with COVID-19 and 232,645 men without COVID-19 reported a significant association of COVID-19 disease with erectile dysfunction, which remained significant upon adjustments for various confounding factors [6]. This study presented a highly compelling evidence regarding the association between COVID-19 and ED. Endothelial dysfunction, testicular damage, cytokine storm, disruption of vascular integrity, and the psychological factors associated with COVID-19 were suggested to account for erectile dysfunction in COVID-19 patients [7]. The SARS-CoV-2 virus has also been found to be present within the penile tissue [8], which explains the direct impact of infection on erectile function.

Eventually, several vaccines were developed to provide immunity against COVID-19 [9]. The acceptance of these vaccines differed greatly across populations, but remained much below 100% in most of the populations. In some countries, the acceptance rate was as low as 30% [10]. Among various presumptive theories related to vaccination hesitancy was the apprehension of adverse effects on fertility, libido, erectile function and overall reproductive health [7, 11, 12]. The effect on the quality of sexual life was the immediate apprehension, while the effect on reproduction was the long-term apprehension related to reproductive health. While there is no concrete evidence regarding the impact of COVID-19 vaccine on erectile function, libido or reproductive health, such trepidation in public translated into low acceptance of COVID-19 vaccines [7, 11, 12]. False beliefs not only restrict vaccine administration, but also hamper the implementation of public health policies aimed at the prevention of infectious diseases and the psychological improvement of society at large aimed at the promotion of rationale and objective approach. Therefore, busting of such myths with scientific evidence is quintessential to promote health policies, social awareness and improved dissemination of health information and mass vaccination drives in situations such as COVID-19.

In the prevailing situation regarding the poor acceptance of COVID-19 vaccine, we undertook the present study to assess the impact of COVID-19 vaccination on sexual functions in males. We collected information about sexual functions using the International Index of Erectile Function (IIEF) questionnaire from Indian men having received COVID-19 vaccine. The study revealed no significant impact of COVID-19 vaccination on male sexual functions, including erectile function, orgasmic function, sexual desire, intercourse satisfaction, and overall sexual satisfaction.

## Methods

### IIEF questionnaire

IIEF (International Index of Erection Function) is a standard questionnaire for clinical assessment of erectile dysfunction [13]. The questions were categorized to examine the five domains of male sexual functions; erectile function, orgasmic function, sexual desire, intercourse satisfaction, and overall satisfaction. A score of 0-5 is awarded to the available options, which makes the maximum score for Erectile function to be 30, for orgasmic function to be 10, for sexual desire to be 10, for intercourse satisfaction to be 15, and for the overall satisfaction to be 10. To determine the effect of COVID-19 vaccination on male sexual functions, we added 15 more questions, one corresponding to each question of the standard questionnaire. In the latter set of questions, the subjects were given an option to choose ‘no change’, ‘negative change’, ‘positive change’ and ‘can’t say’. The full questionnaire is given in Supplementary file 1.

### Subject recruitment

This study was approved by the Institutional Human Ethics Committee of Shri Mata Vaishno Devi University, Jammu (SMVDU/IERB/22/05) and Guru Ghasidas Vishwavidyalaya, Bilaspur (GGV/IEC/2022/02/006). The inclusion criteria were sexually active males in the age-group of 20-50 years who had recently received COVID-19 vaccine. The exclusion criteria were males who were sexually inactive, were below 20 years or above 50 years of age, or were not vaccinated for COVID-19. For data collection, we chose four collection hubs located in the extreme north (Jammu), mid North (Lucknow), far East (Guwahati) and the central (Chhattisgarh) parts of India. From these centres, the link to the Google form was randomly distributed to potential participants by E-mail or smartphone chat apps. Similarly, hard copies of the form were distributed to the local people who were not internet friendly. All data thus collected were collated in an excel sheet for further analysis at the centre of investigation. Along with their responses to the standard questionnaire, we collected E-mail, age and the month of vaccination of the participants. The responses collected were analyzed to determine the effect of vaccination on male sexual functions.

### Statistical tests and softwares used

The scores were assigned to each response according to the standard IIEF Questionnaire. The score distribution and median scores for five male sexual functions were plotted using ggplot2 package of the ‘R’ software. Kruskal Wallis test followed by Dunn’s multiple comparison post-hoc test was used to compare the median scores of male sexual functions in different age groups using the GraphPad prism software. To evaluate the effect of vaccination, the overall responses were plotted using pie3D function of the ‘R’ package. Subgroup comparisons to assess the effect of age and the duration after vaccination on male sexual functions were undertaken using the Chi square test contingency tables 3*4 and 4*4, respectively.

## Results

### Subjects filtered

We distributed the questionnaire through a circle of friends/collaborators. We collected 505 responses in this study. The participants with no vaccination, no sexual activity and an age above 50 years were excluded. The participants with low average IIEF score were considered as ED patients and were excluded from the study. After following the strict inclusion criteria, we were left with 465 responses. These responses were taken forward for data analysis.

### IIEF score calculation

**Fig. 1:**
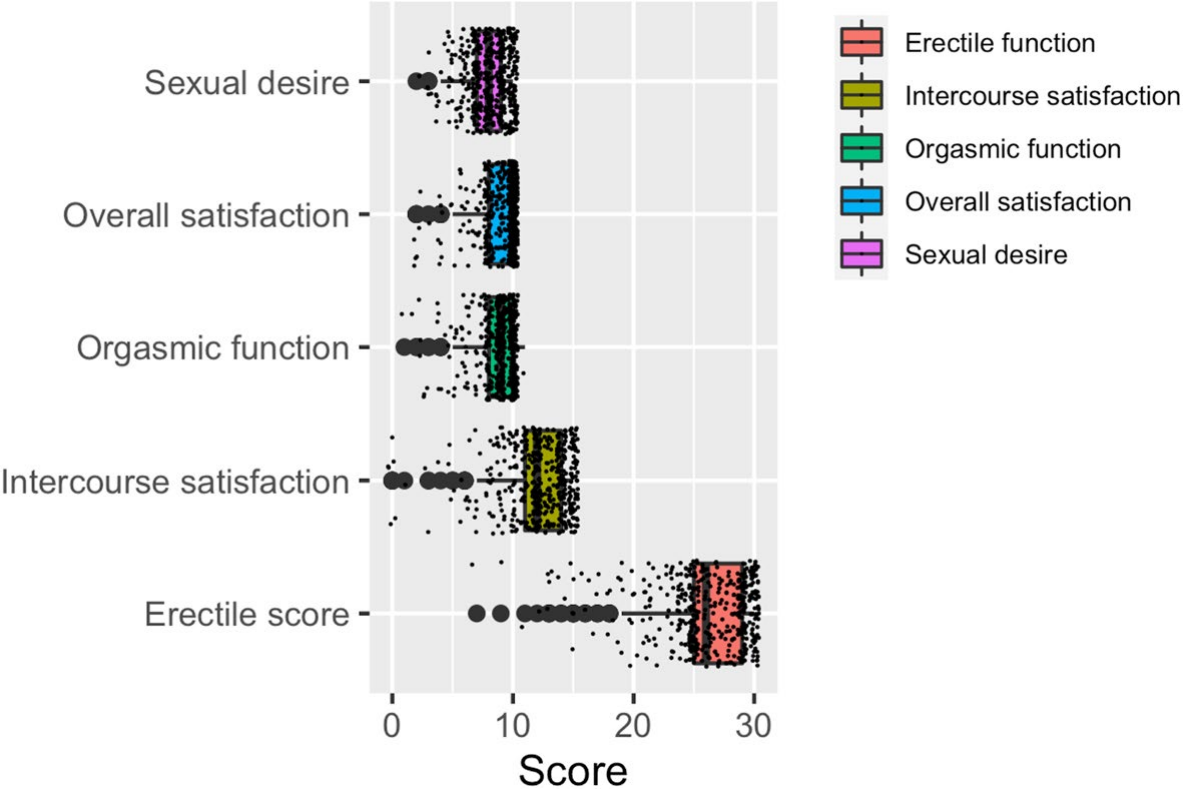
Box plot displaying the distribution of male sexual function scores of the participants

**Fig. 2:**
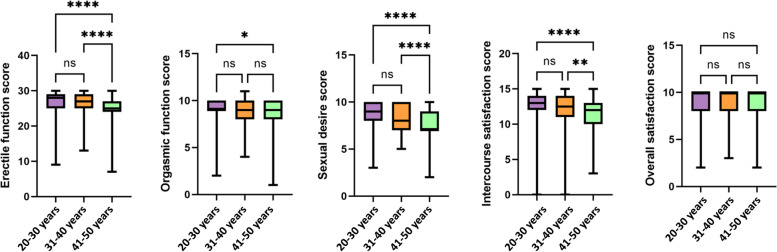
Kruskal Wallis test and Dunn’s multiple comparison analysis of male sexual functions in three age groups (ns = non-significant, * = *p* ≤ 0.05, ** = *p* ≤ 0.01, *** = *p* ≤ 0.001, **** = *p* < 0.0001)

The questions were categorized to examine the five domains of male sexual function: erectile function, orgasmic function, sexual desire, intercourse satisfaction, and overall satisfaction. A score of 0-5 was awarded to the available options, which made the maximum score for Erectile function to be 30, for orgasmic function to be 10, for sexual desire to be 10, for intercourse satisfaction to be 15 and for overall satisfaction to be 10. The average score obtained for each sexual function is presented in Fig. 1. People with low average score, resulting in their classification as ED patients, were excluded. Kruskal Wallis test showed significant differences in male sexual functions across different age groups (*p* < 0.0001 to *p* = 0.0161), except in overall sexual satisfaction score (*p* = 0.2989). This was followed by group-wise comparisons made using Dunn’s multiple comparison test, which showed that the median score for all sexual functions did not differ in 20-30 vs 31-40 years age groups, but erectile function, sexual desire, and intercourse satisfaction differed significantly between 31 and 40 years and 41-50 years age groups. Overall sexual satisfaction did not differ across various age groups (Fig. 2).

### No effect of COVID-19 vaccination on erectile function

**Fig. 3:**
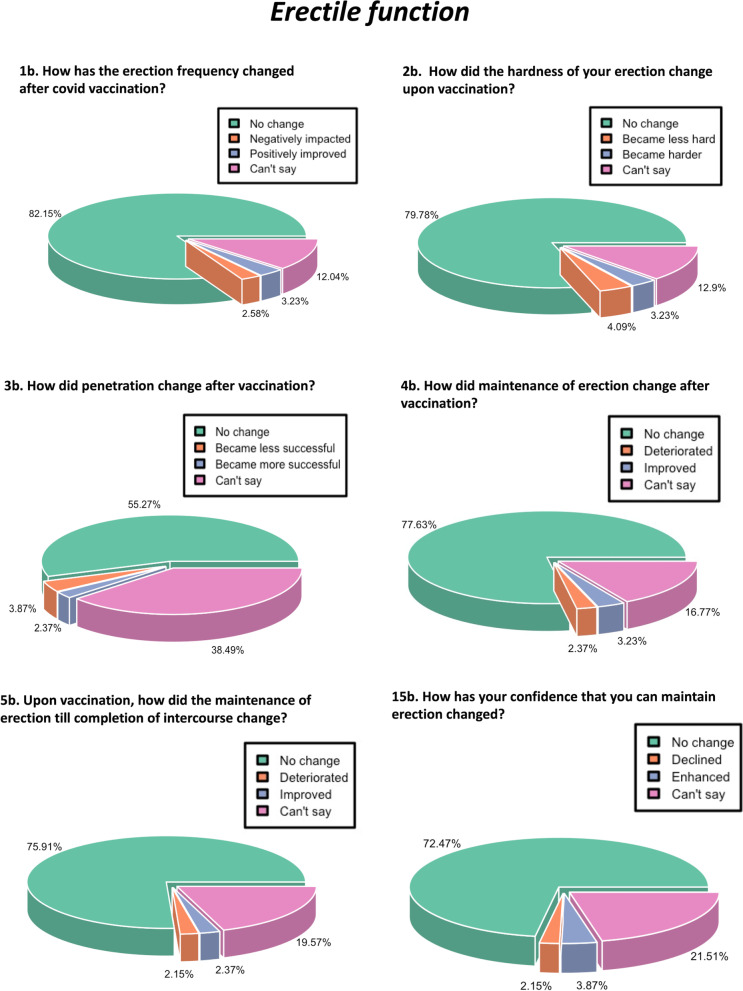
Pie chart representing the percentage response regarding the impact of COVID-19 vaccination on erectile function

**Fig. 4:**
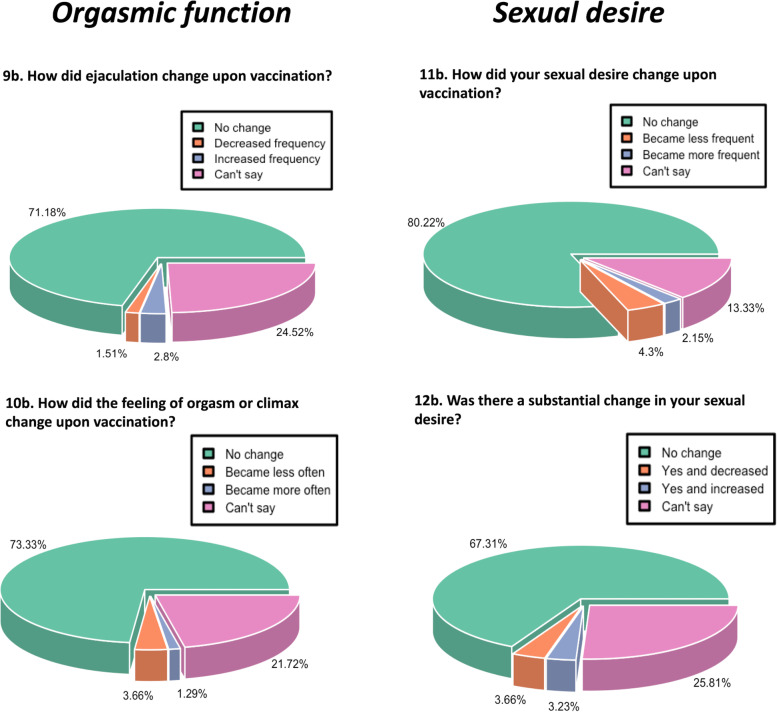
Pie chart depicting the percentage response regarding the impact of COVID-19 vaccination on orgasmic function and sexual desire

**Fig. 5:**
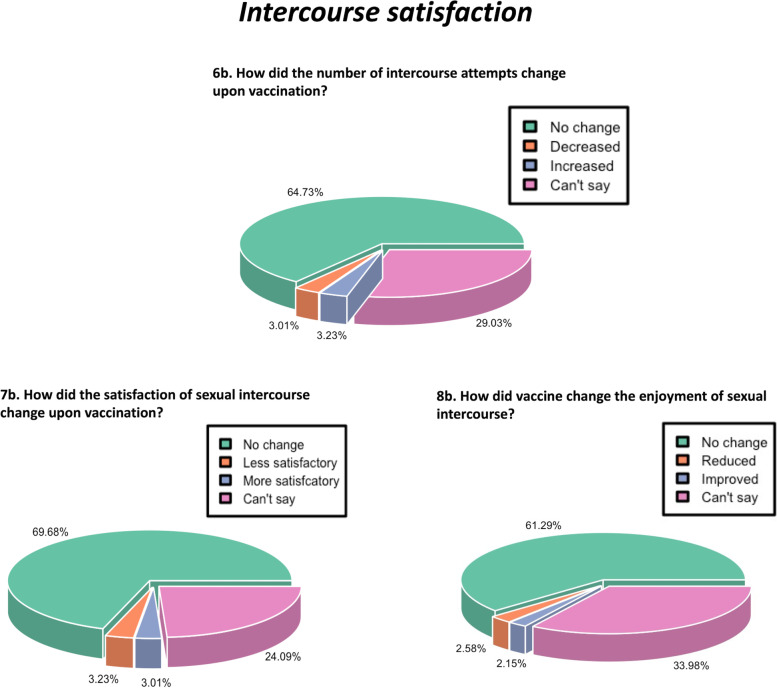
Pie chart depicting the percentage response regarding the impact of COVID-19 vaccination on intercourse satisfaction

**Fig. 6:**
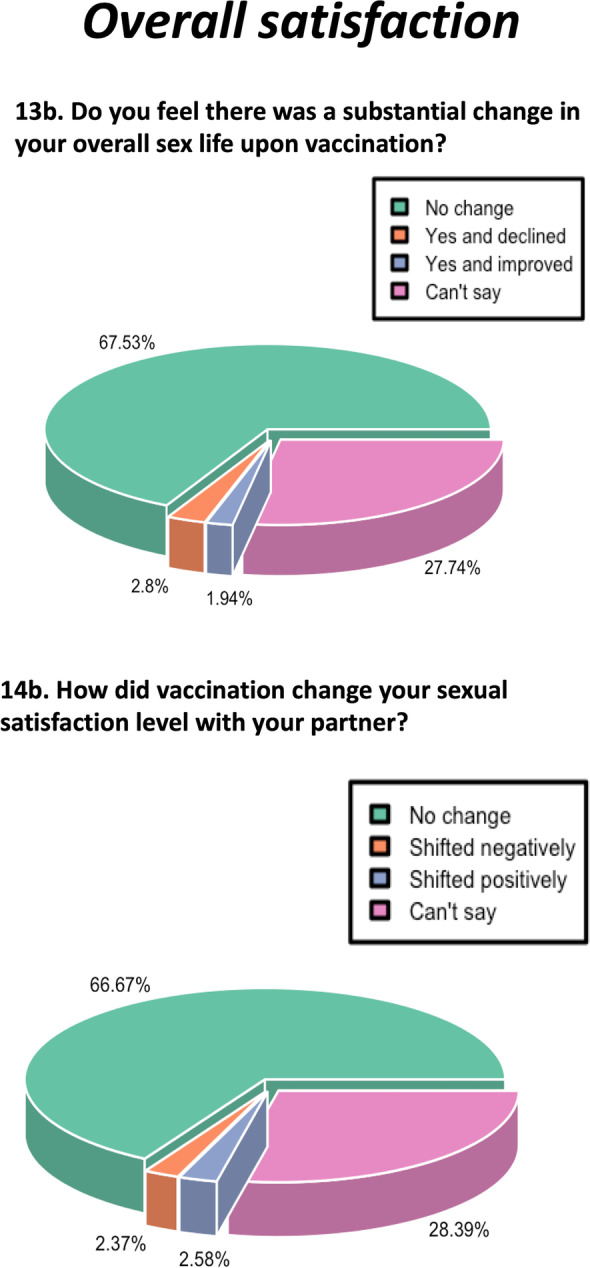
Pie chart depicting the percentage response regarding the impact of COVID-19 vaccination on the overall satisfaction

The effect of vaccination on male erectile function was determined through six multiple choice questions about how erection frequency, hardness of erection, penetration, maintenance of erection on penetration, maintenance of erection till completion, and confidence to maintain an erection have changed upon COVID-19 vaccination. The subjects were given an option to choose ‘no change’, ‘improved’, ‘deteriorated’, and ‘can’t say’. We found that for all male sexual function parameters, the majority of the subjects chose ‘no change’ (Figs. 3, 4, 5 and 6). For erectile function, the responses to six questions were plotted using pie3D function of the ‘R’ software (Fig. 3). We found that 82.15% men reported no change in the erection frequency, 2.58% reported a negative impact, and 3.23% reported a positive impact, and 12.04% reported inability to assess the impact. With regard to the hardness of erection, 79.78% reported no change, 4.09% reported improvement, 3.23 reported a negative impact, and 12.9% expressed inability to score it. Penetrance was reported unchanged by 55.27% of respondents, negatively impacted by 3.87%, positively impacted by 2.37, and 38.49% participants reported inability to score it. With regard to the maintenance of erection, 77.63% reported no change, 2.37% reported a decline, 3.23% reported an improvement, and 16.77% expressed inability to score it. The maintenance of erection till the completion of intercourse remained unaffected in 75.91% individuals, changed negatively in 2.15%, changed positively in 2.37%, and could not be assessed by 19.57% individuals. The confidence to maintain erection till the completion of sexual intercourse remained unchanged in 72.47%, changed negatively in 2.15%, changed positively in 3.87%, and could not be judged by 21.51%.

**Fig. 7:**
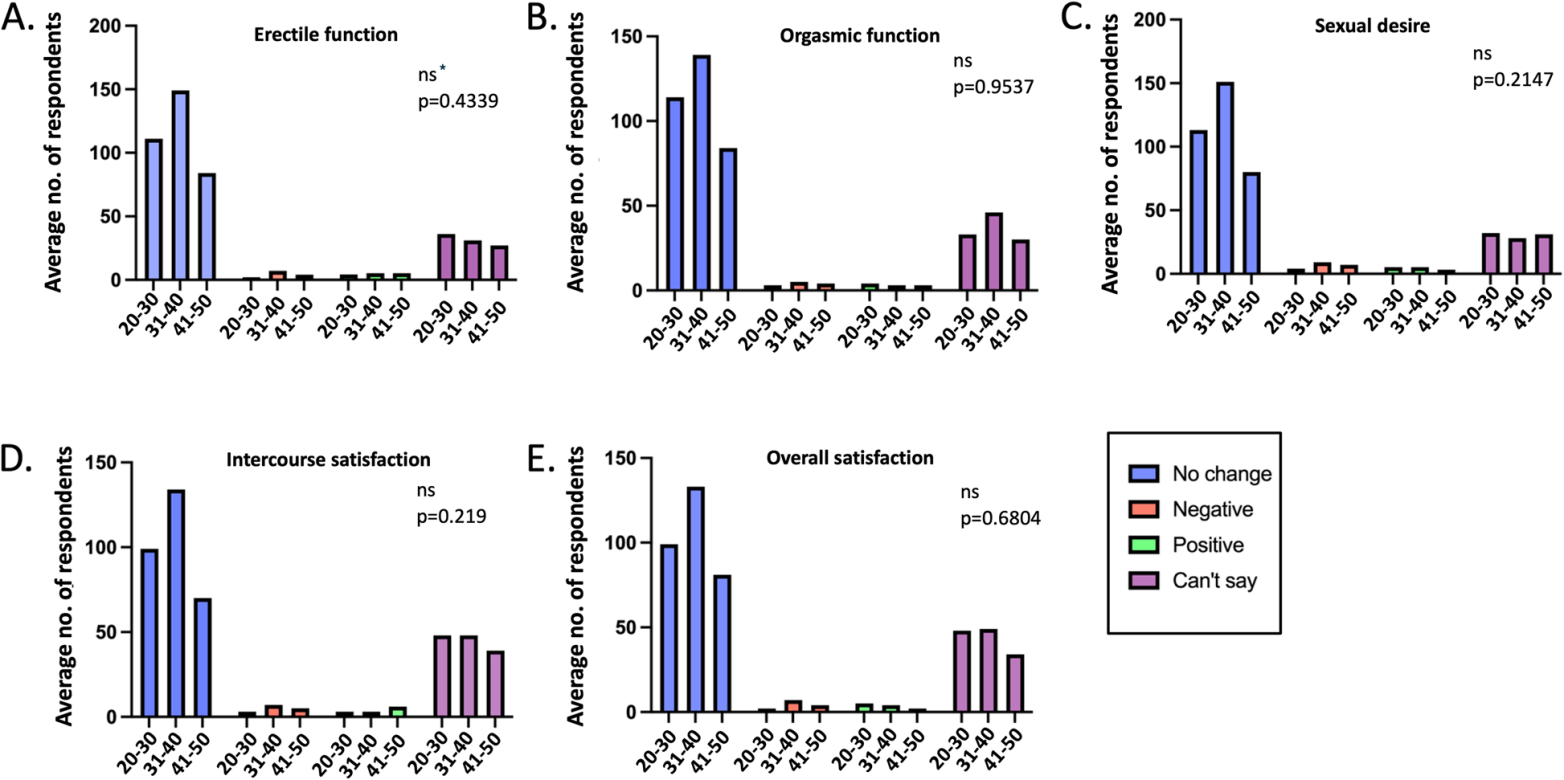
Chi square test comparing the average number of participants with various responses across three age categories. *ns = non-significant

**Fig. 8:**
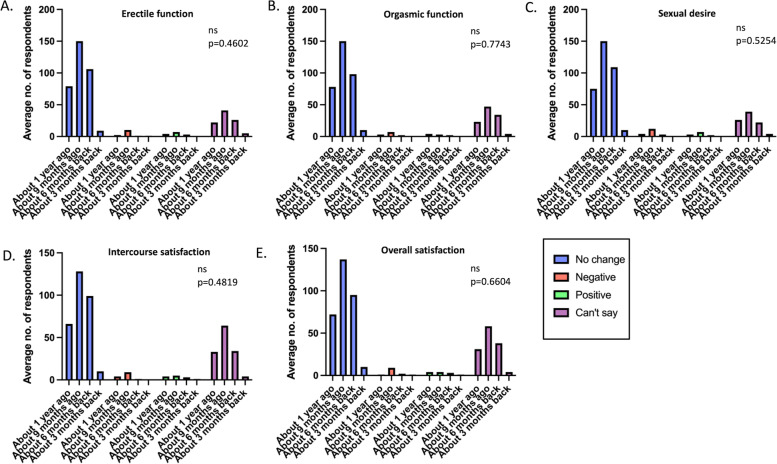
Chi square test comparing the average number of participants with various responses across groups based on the time of vaccination. *ns = non-significant

The responses were also compared according to the age groups and the time of vaccination. The average number of respondents for each option were calculated from the six questions on erectile function and compared using the Chi square test. There was no significant effect on erectile function in any age group (Fig. 7), irrespective of the time of vaccination (Fig. 8), suggesting unlikelihood of short or long-term effects of vaccination (*p* values = 0.4339 and 0.4602, respectively).

### No effect of COVID-19 vaccination on orgasmic function

The effect on orgasmic function was determined through responses to two questions; how has the ejaculation frequency and the feeling of orgasm changed after vaccination? The majority of the participants (71.18%) reported no change in the ejaculation frequency, 1.5% reported a decreased frequency, 2.8% reported an increased frequency, and 24.52% reported can’t say. Similarly, in the case of feeling of orgasm, 73.33% reported no change, 3.66% reported less, 1.29% reported more, and 21.72% reported can’t say (Fig. 4).

The responses were also compared according to the age groups and the time of vaccination to figure out the immediate and long-term effects of vaccination. The average number of respondents for each option were calculated and compared using the Chi square test. There was no significant impact on the ejaculation frequency and orgasmic function, irrespective of the age (Fig. 7) or the time of vaccination (Fig. 8) (*p* values = 0.9537 and 0.7743, respectively).

### No effect of COVID-19 vaccination on sexual desire

A change in sexual desire due to COVID-19 vaccination was determined through responses to two questions: how have the frequency of sexual desire and the level of sexual desire changed upon vaccination? In response to the first question, 80.22% of individuals reported no change in the frequency of sexual desire, 4.3% reported a decrease, 2.15% reported an increase, and 13.33% hit can’t say. Similarly, with regard to the level of sexual desire, 67.31% reported no change, 3.66% reported a decrease, 3.23% reported an increase, and 25.81% hit can’t say (Fig. 4).

The responses were also compared according to the age groups to determine age or the time of vaccination dependent responses to assess the short-term and long-term effects of vaccination. The average number of respondents for each option were calculated and compared using the Chi square test. There was no significant impact on the frequency and the level of sexual desire, irrespective of the age (Fig. 7) or the time of vaccination (Fig. 8) (*p* values = 0.2147 and 0.5254, respectively).

### No effect of COVID-19 vaccination on intercourse satisfaction

Intercourse satisfaction was assessed through three questions: how have the number of sexual attempts, satisfaction frequency, and the level of sexual satisfaction changed upon vaccination? To evaluate the effect of vaccination, their responses to these three questions were noted as ‘no change’, ‘negative effect’, ‘positive effect’, and ‘can’t say’. With regard to the number of intercourse attempts, 64.73% reported no change, 3% reported a decrease, 3.23% reported an increase, and 29.03% hit can’t say (Fig. 5). With regard to the effect on the satisfaction of sexual intercourse, 69.68% reported no change, 3.23% reported a decrease, 3% reported an increase and 24.09% hit can’t say. With regard to the change in the enjoyment of sexual intercourse, 61.29% reported no change, 2.58% reported a reduction, 2.15% reported an improvement, and 33.98% could not assess the impact. The questions on the satisfaction of sexual intercourse got maximum responses as ‘can’t say’, probably due to the difficulty in assessing the overall pleasure of sexual intercourse on a quantitative scale.

The responses were also compared according to the age groups to determine the impact of age and the time of vaccination to evaluate the immediate or long-term effects of vaccination. The average number of respondents for each option were calculated and compared using the Chi square test. There was no significant impact on sexual intercourse satisfaction, irrespective of the age group (Fig. 7) or the time of vaccination (Fig. 8) (*p* values = 0.219 and 0.4819, respectively).

### No effect of COVID-19 vaccination on overall sexual satisfaction

The overall impact on sexual satisfaction was monitored through two questions on overall sex life and sexual satisfaction level with the partner. With regard to the effect on the overall sex life, the majority (67.53%) reported no impact, 2.8% reported a decline, 1.94% reported an improvement, and 27.74% showed inability to assess the impact (Fig. 6). Similarly, with regard to the change in sexual satisfaction with the partner, 66.67% participants reported no change, 2.73% reported a negative shift, 2.58% reported a positive shift, and 28.39% reported inability to score the impact (Fig. 6).

The responses were also compared according to the age groups to determine the impact of age and the time of vaccination to assess the short-term or long-term effects of vaccination. The average number of respondents for each option were calculated and compared using the Chi-square test. There was no impact on the overall sexual satisfaction, irrespective of age (Fig. 7) or the time of vaccination (Fig. 8) (*p* values = 0.6804 and 0.6604, respectively).

## Discussion

COVID-19 disease created havoc during 2020-2021 and prevention was the best solution that left the whole human race with mask on its face. The efforts to develop vaccines started early on and as a result of the dedicated efforts worldwide, a vaccine could be developed within a year of the emergence of COVID-19 disease. Soon after, vaccination started in the United States and other developed countries, with some raising apprehensions regarding its safety amid hurry to roll out a vaccine. Nevertheless, the protection anticipated from vaccination outweighed the unknown side-effects it could pose. The number of deaths in the first and the second waves forced people to go for vaccination, but the vaccination hesitancy due to perceived adverse impact resulted in poor vaccine acceptance across the globe. The fear regarding vaccination has been floated by various socially popular notions or baseless presumptions, resulting in apprehensions and hesitancy to vaccination. This is also affected by various religious beliefs, supported by presumptive adverse impact on sexual health and reproduction, the latter being a major factor in vaccination hesitancy [11]. This has resulted in varying acceptance of COVID-19 vaccine across the world, with very low acceptance in some countries, moderate in others, and good in a few [10].

The viral infections, including SARS-CoV-2, may have temporary impact on the testicular functions, resulting in slight changes in hormone levels and alterations/diversions in molecular signalling, which do not translate into significant changes in sexual function or semen parameters. The reversibility of the mild effects of COVID-19 on the testicular functions was supported by the second set of samples collected after a month, suggesting a temporary effect [14]. Nonetheless, viral infections can result in a prolonged illness and SARS-CoV-2 has even resulted in two months long illness [15]. Some of the COVID-19 effects have been seen even months after the active infection was eradicated [15]. In such cases, some temporary impact on almost every organ, even as distant as testes, can be expected. The SARS-CoV-2 virus was predicted to affect male reproductive functions and semen parameters as the ACE2 receptor required for virus penetrance is expressed in the male germ cells as well [16]. The first report from China about the presence of SARS-CoV-2 in the semen samples of COVID-19 patients raised apprehensions regarding the impact of COVID-19 on testicular functions [17]; however, the presence of virus in semen was later rejected by many [18–23]. Nevertheless, a significant temporary decline in the semen parameters of COVID-19 patients was confirmed by several studies [14, 24–27]. This has created fear in the minds of people regarding the long-term impact of COVID-19 disease on reproductive health. Unfortunately, the fear of infection resulting in an impact on sexual functions, without considering its reversibility post-infection, has been extended to vaccination as well. The fear regarding the negative impact of vaccination on sexual functions or fertility is the major cause of vaccination hesitancy [11]. Regarding the impact of vaccination on fertility, a recent study has shown that there is no effect of mRNA vaccination on semen parameters [28]; however, the possibility of an impact on male sexual functions remained unscored.

In the first such attempt on the impact of COVID-19 vaccination on male sexual functions, we found that a very high number of individuals (average 71%) reported no impact of vaccination on any of their sexual function parameters, including erectile function, orgasmic function, sexual desire, intercourse satisfaction, and overall sexual satisfaction. A significant number of individuals (23.3%) showed inability to score the impact of COVID-19 vaccination on their sexual function parameters. Since a significant impact will be noticeable in some or the other way, we interpret an unclear response (can’t say) in this questionnaire to suggest ‘no significant impact’. This way about 94.3% of individuals suggested no significant impact of COVID-19 vaccination on male sexual functions. Among the remaining (about 6%) individuals, 2-4% reported improvement in most of the sexual functions and the other 2-4% reported a decline is most of the sexual functions, suggesting them to be chance plus and minus variations in reporting. This clears the myths regarding adverse impact of COVID-19 vaccination on male sexual functions.

More than 20 vaccines against viral diseases are already available, of which a set of vaccines is included in the childhood immunization program of various countries. None of these vaccines is known to affect fertility or sexual functions in the adulthood. Among viruses, Mumps is the best-known example of virus induced infertility or reduced testicular volume [27]; however, even Mumps vaccine has not been reported to cause sexual function issues or infertility, despite its introduction about five decades ago. No impact of vaccination on sexual functions is also supported by the fact that they do not cause active illness. Therefore, COVID-19 vaccination does not affect testicular functions, sexual health, libido, and erectile function. In case of COVID-19 vaccines, it is either mRNA, antigen vaccine, or killed virus, and none of the available vaccines uses live attenuated virus. In all of these cases, the live virus does not enter the body or the reproductive organs. Therefore, any effect on reproductive parameters is highly unlikely. This is corroborated by our observations of no impact of COVID-19 vaccination on various aspects of male sexual functions.

The present study had some limitations. It was conducted only in Indian populations; however, we included people from all over the country representing most of the ethnicities available in India; including Caucasians, Indo-Europeans, Asians, south Asians, and Austro-Asiatic populations. The mixed presentation of these ethnicities suggests a highly unlikely different response if such a study is conducted in a different country or on a different ethnic population. It is noteworthy that in the early phase of COVID-19 vaccination, 90% of Indian people received Covishield and 10% received Covaxin. A different vaccine popular in another country, though unlikely to affect sexual functions, may be studied for its impact on male sexual functions. Another limitation was a limited sample size; however, the overall conclusion was that the null hypothesis was not rejected, making it less prone to misinterpretation.

## Conclusion

We conclude that COVID-19 vaccination has no adverse impact on male sexual functions, such as erectile function, orgasmic function, sexual desire, intercourse satisfaction, and overall sexual satisfaction. Taking into consideration the literature, the history of vaccination against viruses, the miniscule and reversible impact of active SARS-CoV-2 infection on testicular functions, and the data collected in this survey, we conclude that COVID-19 vaccination does not cause any adverse impact on male sexual functions. Therefore, the male community should come forward and accept COVID-19 vaccines in order to help the world get rid of the deadly COVID-19 disease that has kept the human population on toes during the last two years. This is important as most of the countries are now starting the vaccination of young children. No evidence of its adverse impact of any kind assures the safety of COVID-19 vaccination for the young or the old alike.

## Supplementary Information

**Additional file 1.**

## Data Availability

The datasets used and/or analysed during the current study are available from the corresponding author on reasonable request.
