# Supplementary material for: COVID-19 vaccination does not affect male sexual functions
Source: Reprod Biol Endocrinol. 2023 Jan 13;21:3. doi: 10.1186/s12958-022-01052-8 (PMC9837459; doi:10.1186/s12958-022-01052-8)
Supplement: Supplementary file 1 — Additional file 1. [file 12958_2022_1052_MOESM1_ESM.pdf]

General information

Some recent reports, which associate SARS-CoV-2 infection with erectile dysfunction (ED) (PMID: 34931145), have created fear among people regarding COVID-19 vaccination. However, there is no study investigating the association between COVID-19 vaccination and ED. Therefore, we planned this survey with an aim to bring a clear picture regarding the impact of COVID-19 vaccine on erectile function, if any.

* Required

1. Email /Name*

____

2. Which age group do you belong to? *

Mark only one oval.

- ( ) 20-30 years
- ( ) 30-40 years
- ( ) 40-50 years

3. Which vaccine have you received?

Mark only one oval.

- ( ) Covishield
- ( ) Covaxin
- ( ) Others
- ( ) None

4. When did you receive first dose of vaccine? *

Mark only one oval.

- About 1 year ago
- About 9 months ago
- About 6 months back
- About 3 months back

Impact of Covid 19 vaccination on erectile function

5. 1a. How often were you able to get an erection during sexual activity?

Mark only one oval.

- No sexual activity
- Almost never or never
- A few times (less than half the time)
- Sometimes (about half the time)
- Most times (more than half the time)
- Almost always or always

6. 1b. How has the erection frequency changed after covid vaccination?

Mark only one oval.

- No change
- Positively improved
- Negatively impacted
- Can't say

7. 2a. When you had erections with sexual stimulation, how often were your erections hard enough for penetration? *

Mark only one oval.

- No sexual activity
- Almost never or never
- A few times (less than half the time)
- Sometimes (about half the time)
- Most times (more than half the time)
- Almost always or always

8. 2b. How did the hardness of your erection change upon vaccination? *

Mark only one oval.

- No change
- Became less hard
- Became harder
- Can't say

9. 3a. When you attempted intercourse, how often were you able to penetrate (enter) your partner? *

Mark only one oval.

- Did not attempt intercourse
- Almost never or never
- A few times (less than half the time)
- Sometimes (about half the time)
- Most times (more than half the time)
- Almost always or always

10. 3b. How did penetration change after vaccination? *

Mark only one oval.

- No change
- Became more successful
- Became less successful
- Can't say

11. 4a. During sexual intercourse, how often were you able to maintain your erection after you had penetrated(entered) your partner? *

Mark only one oval.

- Did not attempt intercourse
- Almost never or never
- A few times (less than half the time)
- Sometimes (about half the time)
- Most times (more than half the time)
- Almost always or always

12. 4b. How did maintenance of erection change after vaccination? *

Mark only one oval.

- No change
- Improved
- Deteriorated
- Can't say

13. 5a. During sexual intercourse, how difficult was it to maintain your erection to completion of intercourse? *

Mark only one oval.

- Did not attempt intercourse
- Extremely difficult
- Very difficult
- Difficult Slightly
- difficultNot
- difficult

14. 5b. Upon vaccination, how did the maintenance of erection till completion of intercourse change? *

Mark only one oval.

- No change
- Improved
- Deteriorated
- Can't say

15. 6a. How many times have you attempted sexual intercourse? *

Mark only one oval.

- No attempts
- One to two attempts
- Three to four attempts
- Five to six attempts
- Seven to ten attempts
- Eleven or more attempts

16. 6b. How did the number of intercourse attempts change upon vaccination? *

Mark only one oval.

- No change
- Increased
- Decreased
- Can't say

17. 7a. When you attempted sexual intercourse, how often was it satisfactory for you? *

Mark only one oval.

- Did not attempt intercourse
- Almost never or never
- A few times (less than half the time)
- Sometimes (about half the time)
- Most times (more than half the time)
- Almost always or always

18. 7b. How did the satisfaction of sexual intercourse change upon vaccination? *

Mark only one oval.

- No change
- More satisfcatory
- Less satisfactory
- Can't say

19. 8a. How much have you enjoyed sexual intercourse? *

Mark only one oval.

- No intercourse
- No enjoyment at all
- Not very enjoyable
- Fairly enjoyable
- Highly enjoyable
- Very highly enjoyable

20. 8b. How did vaccine change the enjoyment of sexual intercourse? *

Mark only one oval.

- No change
- Improved
- Reduced
- Can't say

21. 9a. When you had sexual stimulation or intercourse, how often did you ejaculate? *

Mark only one oval.

- No sexual stimulation or intercourse
- Almost never or never
- A few times (less than half the time)
- Sometimes (about half the time)
- Most times (more than half the time)
- Almost always or always

22. 9b. How did ejaculation change upon vaccination? *

Mark only one oval.

- No change
- Increased frequency
- Decreased frequency
- Can't say

23. 10a. When you had sexual stimulation or intercourse, how often did you have the feeling of orgasm or climax? *

Mark only one oval.

- Almost never or never
- A few times (less than half the time)
- Sometimes (about half the time)
- Most times (more than half the time)
- Almost always or always

24. 10b. How did the feeling of orgasm or climax change upon vaccination? *

Mark only one oval.

- No change
- Became more often
- Became less often
- Can't say

25. 11a. How often have you felt sexual desire? *

Mark only one oval.

- Almost never or never
- A few times (less than half the time)
- Sometimes (about half the time)
- Most times (more than half the time)
- Almost always or always

26. 11b. How did your sexual desire change upon vaccination? *

Mark only one oval.

- No change
- Became more frequent
- Became less frequent
- Can't say

27. 12a. How would you rate your level of sexual desire? *

Mark only one oval.

- Very low or none at all
- Low
- Moderate
- High Very
- high

28. 12b. Was there a substantial change in your sexual desire *

Mark only one oval.

- No
- Yes and increased
- Yes and decreased
- Can't say

29. 13a. How satisfied have you been with your overall sex life? *

Mark only one oval.

- Very dissatisfied
- Moderately dissatisfied
- Equally satisfied & dissatisfied
- Moderately satisfied
- Very satisfied

30. 13b. Do you feel there was a substantial change in your overall sex life upon vaccination? *

Mark only one oval.

- No
- Yes and improved
- Yes and declined
- Can't say

31. 14a. How satisfied have you been with your sexual relationship with your partner? *

Mark only one oval.

- Very dissatisfied
- Moderately dissatisfied
- Equally satisfied & dissatisfied
- Moderately satisfied
- Very satisfied

32. 14b. How did vaccination change your sexual satisfaction level with your partner? *

Mark only one oval.

- No change
- Shifted positively
- Shifted negatively
- Can't say

33. 15a. How do you rate your confidence that you could get and keep an erection? *

Mark only one oval.

- Very low
- Low
- Moderate
- High Very
- high

34. 15b. How has your confidence that you can maintain erection changed? *

Mark only one oval.

- No change
- Enhanced
- Declined
- Can't say

This content is neither created nor endorsed by Google.

Google Forms
